# Improvement in health-related quality of life in osteoporosis patients treated with teriparatide

**DOI:** 10.1186/1471-2474-9-151

**Published:** 2008-11-07

**Authors:** Arthur N Lau, Sammy H Ali, Anna M Sawka, Lehana Thabane, Alexandra Papaioannou, Amiram Gafni, Jonathan D Adachi

**Affiliations:** 1 Department of Internal Medicine, McMaster University, Hamilton, Ontario, Canada; 2 Department of Internal Medicine, University of Toronto, Toronto, Ontario, Canada; 3 Division of Endocrinology and Department of Medicine, University Health Network and University of Toronto, Toronto, Ontario, Canada; 4 Department of Clinical Epidemiology and Biostatistics, McMaster University, Hamilton, Ontario, Canada; 5 Centre for Evaluation of Medicines, St. Joseph's Healthcare, Hamilton, Ontario, Canada; 6 Division of Geriatrics and Department of Medicine, Hamilton Health Sciences and McMaster University, Hamilton, Ontario, Canada; 7 Centre for Health Economics and Policy Analysis, McMaster University, Hamilton, Ontario, Canada; 8 Division of Rheumatology and Department of Medicine, St. Joseph's Healthcare and McMaster University, Hamilton, Ontario, Canada

## Abstract

**Background:**

Individuals with osteoporosis and recent vertebral fractures suffer from pain and impaired health-related quality of life (HRQL). To determine whether patients with osteoporosis treated with teriparatide experienced improvement in HRQL and pain symptoms after several months of therapy.

**Methods:**

We retrospectively studied a sample of osteoporosis patients treated with teriparatide in a Canadian rheumatology practice. We included patients that received teriparatide therapy with baseline and follow-up Mini-Osteoporosis Quality of Life Questionnaire (OQLQ) data. Follow-up data was measured at three or six months. We used a paired Student's t-test to compare baseline and follow-up measurements for each of the questionnaire's ten questions (five domains). Statistical analysis was also repeated to only include patients who suffered a prior vertebral fracture.

**Results:**

57 patients were included in the study, including 47 women. The mean age was 63.8 years (standard deviation 12.1 years). About sixty five percent (37/57) had previously sustained one or more osteoporotic fractures and about 38.6% (22/57) had suffered a prior vertebral fracture. About 44% (25/57) of individuals were taking one or more types of pain medications regularly prior to starting therapy. At follow-up, significant improvements were observed in the OQLQ domains of pain symptoms. This was seen when all patients on teriparatide were included, and also when only patients with prior vertebral fractures were included. There was also an improvement in emotional functioning, relating to fear of falling at 3 months follow-up (p = 0.019). Respondents also reported improvement in the domain of activities of daily living, relating to vacuuming at 6 months follow-up (p = 0.036), and an improvement in the leisure domain, relating to ease of traveling in the prior vertebral fracture population at 3 months follow-up (p = 0.012). However, there was no significant improvement observed in the domains of physical functioning. Participants also reported a decrease in need for pain medications, with 26% (15/57) requiring analgesics at the time of follow-up.

**Conclusion:**

Teriparatide use may be associated with improvements in HRQL in osteoporosis patients, in particular alleviation of pain symptoms. These results were especially evident in patients with a history of vertebral fractures. These findings should be confirmed in larger prospective studies with a suitable control group.

## Background

Osteoporosis is a disease leading to progressive decreases in bone mineral density, decreased bone strength and increased risk of skeletal fractures [1]. Approximately 30% of women will have sustained at least one vertebral fracture by the age of 75 [2]. There are over 700,000 incident vertebral fractures related to osteoporosis each year in the United States [2]. Both clinical and radiographical fractures are associated with an increase mortality rate. One study identified a 16% reduction in expected 5 year survivability [3]. Approximately 75% of patients who present with a clinical vertebral fracture will experience chronic pain [4]. Back pain due to vertebral fractures has a significant impact on osteoporotic patients [4]. The number and severity of vertebral fractures also increases the risk of developing chronic back pain [5]. This has a significant impact on quality of life and functional impairment on the affected patients [6].

Conventional treatments for osteoporosis, including bisphosphonates, selective estrogen receptor modulators (SERMs), calcitonin and estrogen, have been shown to reduce the rate of bone resorption and preserve bone mass [7]. However, none of these have been shown to stimulate new bone formation [7]. Teriparatide [recombinant human PTH-(1–34)] is an agent shown to increase both bone mass and bone strength [8]. In the Fracture Prevention Trial (FPT), teriparatide was shown to increase lumbar spine and femoral neck BMD and decreased fracture risk of both vertebral and non-vertebral fractures in post-menopausal women with osteoporosis [2]. Aside from its effect on BMD, teriparatide also had a positive effect on the non-BMD determinants of bone strength [9].

In the FPT trial comparing the effect of Teriparatide 20 μg/day to placebo in post-menopausal women, the incidence of back pain was 17% in the treatment group, and 23% in the placebo group [8]. Teriparatide's role in preventing back pain in osteoporotic patients was assessed through a meta-analysis of four completed, randomized, double-blinded trials of teriparatide versus a comparator [2]. Nevitt and colleagues reported the teriparatide-treated group had a significant reduction in new or worsening back pain versus comparators (RR 0.73, 95% CI 0.61 to 0.87), over a time period encompassing the clinical trial plus 30 months of post-treatment follow-up assessment [2].

The goal of this study is to determine whether patients in every day clinical practice with osteoporosis, treated with teriparatide, experienced improvement in HRQL and pain symptoms after several months of therapy in a clinic setting. Measuring only pain scores for these patients would be insufficient, because aside from acute and chronic back pain, patients with vertebral fractures also suffer from impaired activities of daily living, anxiety and constant fear about falling and suffering another fracture [10]. A follow-up Mini-Osteoporosis Quality of Life Questionnaire (OQLQ) was used to quantify the patient's pain and impact on quality of life [11]. This is primarily an exploratory study whose sample size is determined by the available data. In addition, this is the first study to compare patient's HRQL data in pre and post-teriparatide therapy.

## Methods

### Study group and inclusion criteria

**Figure 1:**
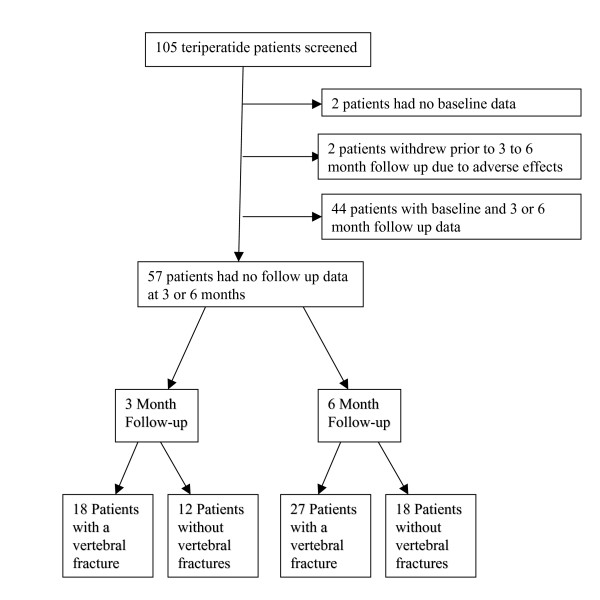
**Enrollment of 105 patients on teriparatide therapy were screened for eligibility. **48 patients were excluded: 2 had not completed baseline questionnaires, 2 withdrew prior ro follow-up due to adverse effects, and 44 had no follow-up data at 3 or 6 months for the most part because they did not have a follow-up appointment at these points.

We conducted a review of osteoporosis patients who had been placed on teriparatide therapy in a Canadian rheumatology practice. Two reviewers abstracted data on demographic information, pain medications, previous fracture history, bone mineral density and health related quality of life (HRQL). In order to be eligible for the study, participants must have completed a baseline and follow-up mini-OQLQ questionnaire prior and subsequent to commencement of teriparatide therapy (at 3 or 6 months) (Figure 1).

### Measurement of health-related quality of life

HRQL was assessed using the mini-OQLQ [11], which was developed for clinical practice as an abbreviated form of the original 30-item OQLQ [11]. As with the original questionnaire, the mini-OQLQ is comprised of five domains: symptoms, physical functioning, emotional functioning, activities of daily living and leisure. The mini-OQLQ has ten items, constructed from the two items with the highest impact in each of the five domains on the original OQLQ. It is a self-administered questionnaire that takes approximately 3 minutes to complete and was designed to be administered in a clinic setting [11]. The OQLQ uses a 7-point scale with a score of 1 representing the worst possible function, and a score of 7 representing the best possible function. A change of approximately 0.5 within each domain is considered to be a clinical relevant difference in quality of life [12,13]. The mini-OQLQ has been validated as a sensitive measure of HRQL in osteoporosis patients with vertebral fracture pain. The application of this tool by Adachi et al. [14] found patients with vertebral fracture had higher scores on all five domains than patients without fracture (Appendix). The Mini-Osteopososis Quality of Life Questionnaire (OQLQ) [11] was completed by the patient at each visit and reviewed with the specialist or nurse clinician. Patients were given the mini-OQLQ prior to initiation of teriparatide and at 3 and/or 6 months follow-up.

### Statistical analyses

As mentioned earlier, this is an exploratory study whose sample size is primarily determined by the available data. The results of this study will provide us with some further insight about the potential effect of teriparatide on health-related quality of life in primary care of patients with osteoporosis which can be explored further in major study. All patients with a follow-up visit at either 3 months or 6 months were compliant with their treatment.

Descriptive data were reported as means (standard deviations [SD]) or median [minimum [min] – maximum [max]) for continuous or discrete variables and count (percentage) for categorical variables. We used the paired Student's T-tests to compare scores on questions of the mini OQLQ prior to and during teriparatide treatment. Analysis was performed comparing the baseline scores to both the follow-up results after 3 and 6 months of therapy. Patients with a prior vertebral fracture were separated and reanalyzed to assess if teriparatide had a greater effect on those with a pre-existing vertebral fracture and/or back pain. We used normal probability plots to assess the Normality assumption. The criterion for statistical significance was set apriori at alpha = 0.05. We used SPSS 12.0 for all statistical analyses.

## Results

### Participant characteristics

The study sample included 57 participants, with 82.5% (n = 47) being female (Table 1). The mean age was 63.9 (SD = 12.1). From the 57 total participants, 45 received their QOL assessment at the 6 month follow up period, and 30 participants were evaluated at the 3-month follow up period. A majority of patients (64.9%) reported that they had previously suffered at least one vertebral or non-vertebral fracture. Vertebral fractures were defined as patients with clinical fractures with x-ray report confirmation. Among the patients evaluated at the 6 month follow-up point, 27 of the 45 patients had suffered a previous vertebral fracture, and 18 of the 30 patients evaluated at the 3 month point suffered a prior vertebral fracture. At baseline, 43.9% of participants (n = 25) reported taking a pain medication for pre-existing back pain. The distribution of different classes of pain medications taken were listed in table 2. The remaining patients with no history of fractures were treated with teriparatide because of severe osteoporosis with subsequent lack of improvement on bisphosphonate therapy.

**Table 1:** Demographic baseline information of patients * Values are mean (standard deviation)

**Variable**	**Number of participants (total = 57)**	***Characteristics of the patients***
Age (years)*	57	63.8 (12.1)

Height (cm) at baseline*	53	160.8 (9.5)

Weight (kg) at baseline*	53	63.3 (15.7)

DXA lumbar spine BMD (g/cm2)*	54	.8298 (.1657)

DXA hip BMD (g/cm2)*	51	.6742 (.1129)

**Female gender**	47	82.50%

**Pain meds at baseline**	25	43.86%

**Vertebral fracture history (one or more)**	22	38.60%

**Multiple fracture history (vertebral or non-vertebral)**	28	49.10%

**History of any type of prior fracture (one of more)**	37	64.90%

**Table 2:** Comparison of pain medication use by patients at time of baseline and follow-up.

	**Before treatment**	**At time of follow-up**
**NSAIDS**	5	4

**Narcotics**	10	6

**Over the counter analgesia**	9	5

**Amitryptyline**	1	0

• Pain medication information was recorded at either the 3 month or 6 month period, whichever was available. If data was available at both follow-up periods, the later one was used.

### HRQL domains and teriparatide

The mini-ORQL can be divided into five distinct domains relating to HRQ: symptoms (Q1, Q2), emotional function (Q3, Q4), physical function (Q5, Q6), activities of daily living (Q7, Q8), and leisure (Q9, Q10). Each question was analyzed individually and grouped according to the domains that they represent (Table 3). The analysis was repeated after only including patients with a prior vertebral fracture (Table 4).

**Table 3:** Baseline and follow-up mean scores of patients at 3 months and 6 months follow-up (includes patients with and withour prior vertebral fractures)

**Domain**	**Question**	**Baseline Mean (SD)-3 Mth patients**	**Baseline Mean (SD)-6 Mth patients**	**3 Month Follow-up (SD)**	**6 Month Follow-up (SD)**	**Sig (2 tailed) 3 Mths**	**Sig (2 tailed) 6 mths**
**Symptoms**	Q1	4.400 (1.850)	3.911 (1.905)	3.533 (1.833)	3.311 (1.607)	0.028	0.039

	Q2	4.233 (1.960)	3.756 (2.112)	3.967 (1.921)	3.200 (1.646)	0.475	0.022

**Emotional Functioning**	Q3	3.633 (2.141)	2.800 (2.052)	3.267 (2.033)	2.578 (1.948)	0.102	0.488

	Q4	3.600 (1.940)	2.889 (1.886)	3.000 (2.034)	2.756 (1.956)	0.019	0.641

**Physical Functioning**	Q5	3.933 (2.016)	3.600 (2.168)	3.967 (1.903)	3.267 (2.093)	0.882	0.092

	Q6	3.867 (2.080)	3.267 (2.230)	3.600 (2.175)	2.911(2.172)	0.428	0.139

**ADLs**	Q7	3.367 (2.632)	3.133 (2.659)	2.933 (2.406)	2.333 (2.558)	0.227	0.036

	Q8	3.467 (2.417)	3.067 (2.416)	2.967 (2.236)	2.489 (2.351)	0.083	0.106

**Leisure**	Q9	3.100 (2.234)	2.378 (2.167)	2.200 (2.091)	2.244 (1.897)	0.001	0.679

	Q10	2.733 (2.586)	2.333 (2.421)	2.500 (2.330)	2.222 (2.245)	0.415	0.628

**Table 4:** Baseline and follow-up mean scores of patients with prior vertebral fractures (3 months and 6 months follow-up)

**Domain**	**Question**	**Baseline Mean (SD)-3 Mth patients**	**Baseline Mean (SD)-6 Mth patients**	**3 Month Follow-up (SD)**	**6 Month Follow-up (SD)**	**Sig (2 tailed) 3 Mths**	**Sig (2 tailed) 6 mths**
**Symptoms**	Q1	5.111 (1.641)	4.37 (2.003)	3.667 (1.782)	3.593 (1.738)	0.005	0.007

	Q2	5.000 (1.749)	4.593 (1.907)	4.444 (1.756)	3.667 (1.710)	0.243	0.009

**Emotional Functioning**	Q3	3.889 (2.398)	2.852 (2.282)	3.667 (2.086)	3.222 (2.136)	0.495	0.401

	Q4	3.722 (2.109)	2.926 (1.817)	3.278 (2.321)	3.259 (2.030)	0.238	0.39

**Physical Functioning**	Q5	5.000 (1.495)	4.111 (2.190)	4.556 (1.756)	3.815 (2.185)	0.149	0.235

	Q6	4.667 (1.782)	3.963 (2.192)	4.333 (1.940)	3.407 (2.275)	0.269	0.13

**ADLs**	Q7	3.944 (1.667)	3.667 (2.815)	3.444 (2.617)	2.963 (2.766)	0.291	0.124

	Q8	4.111 (2.349)	3.667 (2.617)	3.500 (2.282)	2.778 (2.486)	0.135	0.053

**Leisure**	Q9	3.444 (2.332)	2.630 (2.372)	2.500 (2.307)	2.370 (1.844)	0.012	0.571

	Q10	3.556 (2.749)	2.889 (2.651)	3.000 (2.521)	2.519 (2.359)	0.221	0.225

#### Symptoms

The HRQL domain of symptoms showed significant decreases post-teriparatide therapy for both questions that addressed it. Data are expressed as (mean; standard deviation). For patients with 3 month follow-up data, the baseline data for Q1 (4.400; 1.850) was reduced in follow-up data (3.533; 1.833) (p = 0.028), and for patients with 6 month data, the baseline values of (3.911; 1,905) was reduced to (3.311; 1.607) (p = 0.039). Similarly baseline data for Q2 (3.756; 2.112); was lower in the 6 month follow-up data (3.200; 1.646)) (p = 0.022). These results were also evident when only patients with only prior vertebral fractures were included. At 3 month follow up, Q1 decreased from (5.111; 1.641) to (4.444; 1.756) (p = 0.005) and at 6 month follow-up, Q1 decreased from (4.37; 2.003) to (3.593; 1.738) (p = 0.007). Similarly after 6 months, Q2 also decreased from (4.593; 1.907) to (3.667; 1.710) (p = 0.009) for the vertebral fracture group.

#### Emotional functioning

The questions addressing emotional functioning also demonstrated a significant improvement in Q4. The baseline for Q4 (3.600; 1.940) was lower after 6 months of therapy (3.000; 2.034) (p = 0.019). However, this was not evident when only considering patients with prior vertebral fractures. Q3 did not reveal any significant improvement after initiating teriparatide.

#### Physical functioning

Data for the physical functioning domain did not show a significant improvement in either questions, and there was no difference when only considering patients with prior vertebral fractures.

#### Activities of daily living

Question 7 which assessed the difficulty of vacuuming showed a significant improvement at the 6 month follow-up point (3.133; 2.659) to (2.333; 2.558) (p = 0.036) but there was no significant. However, this change was not evident when only considering patients with prior vertebral fractures. Question 8 did not reveal and significant improvement.

#### Leisure

Question 9, which assessed the patient's difficulty in travelling showed a significant improvement after 3 months of therapy in the vertebral fracture patients. The scores decreased from (3.444; 2.332) to (2.500; 2.307) (p = 0.012). Q10 did not reveal any significant improvement after initiating teriparatide.

## Discussion

Vertebral fractures are an important and common cause of morbidity in osteoporotic patients [15]. Vertebral fractures are among the top health conditions accounting for length of hospital stay, and added significantly to the length of stay to patients admitted for other medical problems [16]. Aside from the physical limitations suffered by these patients, chronic back pain has a significant impact on the patient's quality of life [17]. Patients suffering from vertebral fractures often have impaired physical functioning, limited activities of daily living, limited leisure and recreational activities, and significant emotional distress [11]. The use of teriparatide in the treatment of postmenopausal osteoporosis revealed a decrease in the risk of both vertebral and non-vertebral fractures, along with a significant increase in vertebral, femoral neck, and total body-bone mineral density [8]. The goal of our study is to evaluate the effect of teriparatide treatment on the risk of back pain and health related quality of life in osteoporotic patients in a clinical practice setting. Studies in the past have investigated the effect of teriparatide on back pain risk in a randomized control trial setting [2]. Nevitt and associates' systematic review identified five randomized clinical trials (RCTs) that evaluated prevention of back pain in teriparatide treated osteoporotic patients. In contrast, our study investigates patients with pre-existing back pain, and we measured the improvement in pain severity and effect on quality of life after initiating teriparatide therapy. Also, the incidence of back pain was not the primary outcome in any of these studies, with new vertebral fractures (n = 1) or changes in bone mineral density (n = 4) as the primary outcomes [2]. Back pain data were collected through spontaneous reporting by patients as adverse events. The conclusion of the meta-analysis was that patients randomized to teriparatide had a reduced risk of new or worsening back pain compared to patients randomized to placebo or anti-resorptive therapies [2]. RCTs have several disadvantages. Although RCTs are able to demonstrate the efficacy of a therapy, it has certain limitations in demonstrating the therapy's effectiveness in a real world patient population. Firstly, patients in RCTs represent a very homogeneous patient population that may not reflect the target patient population. These patients are also self selected, therefore they have higher likelihood of having a high compliance rate to treatment [18].

Analysis of our HRQL data in our patients' pre and post-teriparatide therapy revealed statistically significant improvements predominantly in the domain of symptoms, but there were also improvements in the domains of emotional functioning, ADLS, and leisure. The symptoms domain inquired about discomfort/distress related to pain, while the emotional functioning domain addressed the patients' fears of two major complications, fractures and falling. A reduction in the symptoms domain indicates that patients who received teriparatide therapy went on to experience less pain than prior to therapy. In addition, there was a decrease in patients requiring analgesics after treatment (a drop from 44% to 26% (Table 4). Analgesia use was identified using patient information questionnaires. Based on the data collected, there is a suggestion that teriparatide use may decrease improve the need for analgesia. However, many of the patients who completed the questionnaire did not indicate the specific dosage of the analgesia they were taking. Therefore, we were unable to assess if there was a decrease in frequency or potency in analgesia use. An improvement in emotional functioning scores suggests that patients treated with teriparatide had less fear of falls than they experienced before receiving the medication. Therefore patients had less pain discomfort at both three to six months after being started on teriparatide, and these trends were also seen when only patients with a history of vertebral fractures were included. There are several limitations to our study, including missing data resulting in exclusion of some participants, and lack of a control group.

## Conclusion

In conclusion, previous studies have demonstrated that teriparatide therapy results in decreased fractures and pain symptoms in patients with osteoporosis, and may be particularly beneficial in patients with a history of vertebral fractures. There was a greater statistical significance in the vertebral fracture population than in the total population. Patients also appeared to benefit quickly after therapy was initiated, as a significant difference was evident even at the 3 month follow-up period for certain domains in the HRQL survey. In this study, we have confirmed that patients with osteoporosis treated with teriparatide experience improvements in pain symptoms. Furthermore, certain aspects of emotional functioning, activities of daily living, and leisure activities appears to improve with therapy. Our findings need to be validated in a larger prospective study with a suitable control group.

## Competing interests

Sources of support: This study was an unfunded and was a project undertaken by Arthur Lau and Sammy Ali at McMaster University. The authors declare that they have no competing interests.

Dr. Adachi has received consulting fees from Amgen, Astra Zeneca, Eli Lilly, Glaxo Smith Kline, Merck, Novartis, Pfizer, Procter & Gamble, Roche, Sanofi Aventis, Servier.

## Authors' contributions

JDA conceived of the study, and participated in its design and coordination and helped to draft the manuscript. ANL was the principal author of the manuscript, and carried out the data collection and statistical analysis. SHA helped to draft the manuscript and shared in the data collection process. AMS, LT, AP and AG aided in the drafting of the manuscript.

## Appendix: Shortened Osteoporosis Quality of Life Questionnaire

1) How much distress or discomfort have you had because of pain in the last two weeks?

2) How much distress or discomfort you had in last two weeks because it had been painful to stand for a long time

3) How often in the last two weeks have you felt afraid of fractures?

4) How often in the last two weeks have you felt afraid of falling?

5) How difficult has it been for you to lift things in the last two weeks?

6) How difficult has it been for you to carry things in the last two weeks, because of back problems due to osteoporosis?

7) How difficult has it been for you to vaccum in the last two weeks?

8) How difficult has it been for you to housework in the last two weeks?

9) How difficult has it been for you to travel in the last two weeks?

10) How difficult has it been for you to take the type of vacation or holiday you enjoy because of your back problems due to osteoporosis

## Pre-publication history

The pre-publication history for this paper can be accessed here:


